# 

**Published:** 1899-12-23

**Authors:** 


**Notice to Advertisers and Subscribers.**

All Advertisements, Orders for Copies of The Hospital, and Business Communications should be addressed to **THE MANAGER (Not to the Editor)**, The Hospital, The Hospital Building, 28 & 29, Southampton Street, Strand, London, W.C.

The Cost of Subscription, post free, is as follows:—Per week, 2½d.; per quarter, 2s. 8d.; per half-year, 5s. 3d.; per annum, 10s. 6d. Foreign:—Per annum, 15s. 2d., payable, in all cases, in advance.

**NOTICE.—Vol. XXVI. of "The Hospital" (April, 1899, to September, 1899), in handsome cloth binding, is now on sale at the Office of this Journal, price 7s. 6d. Cases for binding the Numbers contained in this Volume 1s. 6d. Index to Vol. XXVI., 2½d., post free.**

**The Monthly Part for January will be ready on 1st January, 1900. Price 6d., by post 8d.**

### NOTES ON BOOKS.

Mr. B. G. Jenkins, F.R.A.S., has sent us a copy of his "Weather Chart for 1900" (London: R. Morgan, 65, Weston Street, Upper Norwood). This gives in a series of curves the "Teluric Curve," the "Forecast Barometer," and the "Forecast Thermometer" throughout the year. In addition to this a general outline is given of the probable weather in each month. The chart is published in two sizes; the large one, 25 in. by 10 in., price 6d., and the small one, 12 in. by 5 in., price 1d. Each of them post free for ½d. extra. If they are to be depended on it is clear that the purchaser gets his money's worth. Forecasting of the weather is always a matter of interest, and in order that we may judge Mr. Jenkins by his works we append his forecast for January, 1900. "Probable weather, January: Unsettled round 4th, high barometer with fog round 8th, and for ten days round 21st. Stormy with gales and snow round 13th and at the close. Temperature: Very cold on 4th, and for ten days round 18th. Mild about 9th and 26th. Cold with snow at the close. Rain: Below the average, chiefly about 5th, round 13th, and at the close. . . . Prevailing winds: S.W., S.E., N.E., southerly winds predominating." We may add that Mr. Jenkins seems to be affected with a sort of meteorological hypermetropia—he sees most distinctly at long ranges. There is a certain caution in his predictions for the earlier months. Things happen "about the 1st" or "round the 17th," but when we get further on we become more definite. In October we are told that there will be gales during three weeks, "probably with snow on the 20th." But that is nothing to the forecast for December, under which month we find the notes "Snow on the 7th and 16th," "Snow on 23rd." These long-range prophecies are, we must confess, a little trying to one's faith, even in Mr. Jenkins. Still a comparison of the real with the prophetic will not be without interest.

### BOOKS RECEIVED.

Baillière, Tindall, and Cox.

"A Synopsis of the British Pharmacopœia," 1898. By H. Whippell Gadd and C. G. Moor, M.A., F.I.C. Fourth edition.

T. Fisher Unwin.

"Hermann Ludwig Ferdinand von Helmholtz." By John Gray McKendrick, M.D., LL.D., F.R.S.S., L. and E.

"The Private Nurse." By Jessie Holmes.

Student Volunteer Missionary Union.

"The Healing of the Nations: A Treatise on Medical Missions." By J. Rutter Williamson, M.B.

Simpkin, Marshall, Hamilton, Kent, and Co.

"Common Sense Health Reform." By T. Thatcher.

J. B. Lippincott and Co.

"System of Diseases of the Eye." Edited by William F. Norris, M.D., and Charles A. Oliver, M.D. Volume IV.

Sampson Low, Marston, and Co.

"Twentieth Century Practice of Modern Medical Science." Edited by Thomas L. Stedman, M.D.

### The Student of Medicine.

### APPOINTMENTS.

G. E. Bloxam, M.R.C.S., L.R.C.P., appointed Medical Officer to the Western Dispensary, Bath. F. Butterfield, M.B.Lond., appointed Junior House Surgeon of the Blackburn and East Lancashire Infirmary. A. H. Evans, M.D., B.S.Lond., F.R.C.S.Eng., appointed Senior Surgeon to the East Dispensary, Liverpool. A. A. Gunn, M.B., Ch.B.Edin., appointed Senior House Surgeon of the Blackburn and East Lancashire Infirmary. J. B. Hellier, M.D.Lond., appointed Honorary Obstetric Physician to the Leeds Infirmary. J. J. Hopkins, L.&L.M.R.C.P.I., L.&L.M.R.C.S.I., J.P., appointed Medical Officer and Medical Officer of Health to the Castlebar No. 1 District. T. N. Kelynack, M.D.Vict., M.R.C.P.Lond., appointed Assistant to the Professor of Medicine, the Owens College, Manchester. H. G Nicholson, M.R.C.S., L.S.A., appointed Honorary Medical Officer to the Queen Adelaide Dispensary, Bethnal Green. J. N. Paterson, M.B., C.M., F.R.C.S.E., appointed Assistant Ophthalmic Surgeon to the Edinburgh Royal Infirmary. R. Pugh, M.B., Ch.B., appointed Senior Assistant Medical Officer at the Birmingham City Asylum. E. C. Ryall, M.R.C.S.Eng., &c., appointed Surgical Registrar to the Westminster Hospital, and Demonstrator of Physiology in the Westminster Hospital Medical School. C. E. Smith, M.B., Ch.B.Edin., appointed Junior Assistant House Surgeon to the Royal Devon and Exeter Hospital, Exeter. St. J. Stanwell, M.B.Edin., M.C., M.R.C.S., appointed Surgeon to the Stamford and Rutland General Infirmary, and Surgeon to the Post Office at Stamford. A. L. Whitehead, M.B.Lond., B.S., M.R.C.S.-Eng., appointed Honorary Ophthalmic and Aural Surgeon to the Leeds General Infirmary. J. Wigg, L.R.C.P.Lond., appointed District Medical Officer for Ward 2 of the St. Pancras Parish.

Royal 8vo., illustrated by a series of Original Photo-micrographs and many Illustrations in the Text, cloth gilt, 7s. 6d. net,

### THE MENOPAUSE AND ITS DISORDERS.

**With Chapters on Menstruation.**

By A. D. LEITH NAPIER, M.D., M.R.C.P., late Editor of the *British Gynæcological Journal.*

"Of great practical use to the general practitioner as well as to those more specially engaged in gynæcological work."—*The Hospital.*

London: The Scientific Press, Ltd., 28 & 29, Southampton Street, Strand, London, W.C.

### HOVIS BREAD

Is always nice. Made by most Bakers. Local agents' addresses sent on application by HOVIS, Ltd., Macclesfield.

### Hospitals, &c., Urgently Needing Annual Subscriptions, Donations, and Legacies.

### LONDON HOSPITAL, E.

**The Committee appeal for £40,000 a-year from VOLUNTARY CONTRIBUTIONS.**

The number of IN-PATIENTS treated in 1897 was 11,146
" OUT-PATIENTS " " 161,033
Total number of Patients treated at the Hospital—172,179

**FUNDS ARE URGENTLY NEEDED.**

**Annual Subscribers are entitled to Tickets.**

Thoroughly-Trained Private Nurses to be had immediately on application to the Matron.

Honble. SYDNEY HOLLAND, *Chairman.* G. Q. ROBERTS, *House Governor.*

**THE UNIFORM SYSTEM OF ACCOUNTS, AUDIT, AND TENDERS, for Hospitals and Institutions, with Certain Checks upon Expenditure; also the Index of Classification.** Compiled by a Committee of Hospital Secretaries, and adopted by a General Meeting of the same, 18th January, 1892. And certain Tender and other Forms for securing Economy, By Sir HENRY BURDETT, K.C.B. Demy 8vo, profusely illustrated with Specimen Tables, Index of Classification, Forms of Tender, &c., cloth extra, 6s.

**ACCOUNT BOOKS FOR INSTITUTIONS, designed in accordance with the Uniform System of Accounts for Hospitals and Institutions.** By Sir Henry Burdett, K.C.B. Being a complete set of Account Books ruled in accordance with the Uniform System adopted by the Metropolitan Hospital Sunday Fund.

*(Full Description of Books and Prices sent post free on application.)*

London: The Scientific Press, Ltd., 28 & 29 Southampton Street, Strand, W.C.

Accouchement Requisites. Ambulance Cases.

### NOW READY,

A REPRINT OF

### A NURSING HANDBOOK

TOGETHER WITH S. MAW, SON, AND THOMPSON'S

### COMPLETE CATALOGUE OF NURSING REQUISITES.

The above work is now republished at a cost of 1s. per copy. Messrs. S. M., S., and T. have been compelled to make this charge on account of the extreme popularity of the work and the great expense incurred in its production.

***Post Free on receipt of 1s.***

### S. MAW, SON, and THOMPSON,

7 to 12, ALDERSGATE STREET, LONDON, E.C.

